# Clinical Lectures on Diseases of the Eye

**Published:** 1865-09

**Authors:** E. L. Holmes

**Affiliations:** Chicago, Lecturer on Diseases of the Eye and Ear in Rush Medical College, and Surgeon of the Chicago Charitable Eye and Ear Infirmary


### CLINICAL LECTURES ON DISEASES OF THE EYE.

By E. L. HOLMES, M. D., of Chicago,
*Lecturer on Diseases of the Eye and Ear in Rush Medical College, and Surgeon of the Chicago Charitable Eye and Ear Infirmary.*

### DISEASES OF THE RETINA AND OPTIC NERVE.

GENTLEMEN:

Referring you to standard works on anatomy for a description of the optic nerve posterior to the globe, I will simply call your attention to that portion of it which will be of most importance in your investigations of diseases involving the nervous apparatus of the eye. This portion is the papill of the optic nerve. After the nerve has passed through the sclerotic and choroid, as described in a recent lecture, it becomes a thin membrane spread over the concave surface of the choroid. The end, as it were, of the nerve, however, can be seen as a well-defined disk, in many cases slightly elevated above the surrounding nervous tissue. This disk is scarcely larger than the head of a pin, yet, when examined with the aid of the ophthalmoscope, it appears much larger.

The retina is a thin transparent membrane, varying in thickness from 1-20 to 1-10 of a line, and may be divided into seven distinct layers. The middle layer is composed of the nervous fibres of the optic nerve, radiating in all directions towards the anterior portion of the eye. The other layers are composed of granules, ganglionic cells, and so-called bacilli and coni. These various elements, as also the separate fibers of the optic nerve, are united by an exceedingly delicate areola tissue, which plays an important part in inflammation.

The vessels of the retina, consisting of branches of the central artery and vein of the optic nerve, branch upwards and downwards, as you can see by close inspection of this preparation of the posterior hemisphere of the globe, and are represented with the papilla in this beautiful diagram of Jaeger.

A line and a half to the temporal side of the optic papilla, at a point corresponding to the posterior end of a line carried through the centre of the cornea and of the globe itself, to the retina, is the macula lutea, the point of most distinct vision.

The retina is held very loosely in connection with the choroid, except at its anterior edge, where it loses its nervous structure and becomes a delicate, transparent, yet quite strong membrane, firmly attached to the ciliary processes. It here divides into two lamellæ, one of which is united to the anterior and the other to the posterior surface of the capsule of the crystalline lens, near its periphery, and is called the zonula of Zinn.

The discussion of the functions of the retina is one of the most interesting subjects in physiology, but would alone require several lectures. I must therefore refer to the chapters on vision in the best works on physiology.

There is a peculiar change in the retina of aged persons, which, like the turning gray of the hair, can scarcely be called a disease, and which yet causes to a certain extent the dimness of vision observed in advanced life. This consists in the deposit among the intranervous areola tissue of greyish colored granules and also of minute glass-like particles, both of which in some cases can be seen by means of the ophthalmoscope in the mottled appearance of the retina.

The diseases of the retina most common, and therefore most important for your consideration, are the different phases of inflammation. The three cases before you to-day are examples of retinitis in different forms, one from Bright's disease, another from syphilis, and the other obscure in its origin. The precise manner in which the inflammatory process goes on is not understood. It is, however, well established that the areola tissue which holds the different elements of the retina and optic nerve together is first implicated. The cells of this tissue enlarge, new cells are formed, and a yellowish exudation takes place. The final results of these changes depend upon their duration and violence and extent of surface implicated. The layers of the retina nearest the choroid seem most liable to inflammation. Some time may elapse before the vessels of the retina which are in the layers nearer the vitreous humor become covered with exudation to such an extent that they are rendered invisible in an ophthalmoscopic examination. The inflammation may be uniform over the whole retina and papilla of the optic nerve, or it may be circumscribed within very narrow limits. Retinitis is often associated, either as a cause or effect, with choroiditis. The exudation is often mingled with a large number of pigment cells, which can be seen with the ophthalmoscope as irregular brown or black patches, and are frequently found in and around the papilla and around circumscribed portions of the retina affected with inflammation.

Not unfrequently minute points of extravated blood will be observed in the retina. One of the most common abnormal appearances is caused by the presence of minute radiating lines, extending from the centre of the papilla to the ora serrata, obliterating the line of demarcation between the retina and papilla.

The effects of inflammation upon the structure of the retina are various. The disease may be of short duration, terminating in the return of perfect vision. In other cases, it may cause atrophy of the nervous tissue; or it may cover the nervous fibres, with an organizable lymph, which may never be absorbed, and yet, upon the removal of the eye, the substance of the retina within this lymph, may be found to retain its microscopic appearances intact. The inflammation often produces atrophy of the papilla, and a softening of the lamina cribrosa of the sclerotic, in consequence of which changes, this portion of the globe cannot withstand, even the normal pressure of the nitra ocular fluids, and cupping of the papilla is the result.

Inflammation of the optic nerve may lead, as in that of the retina to atrophy from disarranged nutrition. As in other organs of the body, inflammation of the retina and optic nerve may lead to falty generation.

As the result of retinitis, much oftener as a result of choroiditis, sometimes suddenly and without any known cause the retina may become detached from the choroid, either in small circumscribed portions or over its whole extent.

The causes of retinitis are often very obscure. Injuries, imprudent use of the eyes, syphilis, scrofula and certain affections of the heart, lungs and kidneys, especially Bright's disease, excessive lactation are each sufficient in different cases to induce inflammation of the nervous structure of the eye.

The course of this class of diseases is usually slow, and subacute in character. Acute inflammation, resulting in suppuration is not common. Under suitable treatment, the disease may recover, but it often resists the effects of all known therapeutic agents.

The principal symptom of inflammation of the retina and optic nerve is dimness of vision without any appearance of opacity of the refracting media. If a portion of the retina or a few fibres only of the optic nerve are affected, there will be a dark spot constantly in the field of vision, in the part corresponding to the affected portion. This last symptom is often indicative of detachment of the retina from the choroid. There is generally little pain in uncomplicated inflammation of the retina and optic nerve. The patient is more liable to complain of unpleasant flashes of different colored light. The iris is usualy normal in appearance, and the pupil movable, although more sluggish than in health. Congestion of the conjunctiva and submucous membrane is seldom observed.

The only means of detecting the objective symptoms is by means of the ophthalmoscope. From my previous remarks and from examining at the plates of Jaeger and Liebreich, you already know what these symptoms are. Some of you have had opportunities of observing with ophthalmoscope extravasated blood, detached portions of the retina floating in the vitreous humor, abnormal conditions of the vessels, the presence of pigment, and yellow exudation and the changes peculiar to the papilla of the optic nerve.

The leading principles upon which you must base your treatment are comparatively few.

The eyes should be kept in perfect darkness for ten or twelve days, rather by means of a light, yet efficient bandage, than by confining the patient in a dark room. The eyes should be uncovered once daily in a dark room, for the purpose of bathing.

Local bleeding by means of the artificial leech has been used with great advantage in some cases.

The use of Bichloride for mercury, Iodide of Potash, Permanganate of Potash, Bromide of Ammonium, may be tried according to circumstances.

Strict attention should be directed to the condition of all the organs of the body, and appropriate remedies applied in case of their unhealthy action.

Retinitis dependent upon syphilis, even when the vessels and papilla are to a great extent concealed from view, may sometimes be wholly removed by specific remedies and vision restored.

Even when existing as a complication of Bright's disease, with serious amaurotic symptoms, vision may be much improved temporarily by means, which modify the condition of the kidneys.

It should be remembered that treatment is most frequently followed by favorable results, when commenced in the earlier stages of the disease. If of long duration, the disease must be considered as very grave.

